# Contribution of Blood Orange-Based Beverages to Bioactive Compounds Intake

**DOI:** 10.3389/fchem.2018.00374

**Published:** 2018-08-29

**Authors:** Fabio Licciardello, Elena Arena, Valeria Rizzo, Biagio Fallico

**Affiliations:** ^1^Dipartimento di Scienze della Vita, University of Modena and Reggio Emilia, Modena, Italy; ^2^Dipartimento di Agricoltura, Alimentazione e Ambiente, University of Catania, Catania, Italy

**Keywords:** commercial beverages, water-based, fruit-based, ascorbic acid, anthocyanins, hydroxycinnamic acids, red orange juice

## Abstract

The study aimed at screening the levels of ascorbic acid, hydroxycinnamic acids (HCAs) and total anthocyanins in commercial beverages containing blood orange juice (BOJ), estimating the contribution of these products to the intake of health-promoting compounds and at discussing the actual value of the products on a price/bioactive level basis. Results demonstrate that the reference to BOJ in many beverages is misleading, as inferred from the very low bioactive levels observed. These beverages, in label should refer as “water-based” instead of “fruit-based beverage.” Accordingly, the intake of bioactives from BOJ-based beverages, with the exception of ascorbic acid used in the most cases as preservative, is often very low. The intake of bioactive components from blood orange consumption is much higher compared to the consumption of BOJ-based beverages, hence the consumption of blood oranges and 100% BOJs should be preferred in order to guarantee the intake of a rich pool of bioactive compounds. Finally, the market value of BOJ-based beverages is not correlated with their real nutritional value: the relative unitary cost of the three classes of bioactive compounds considered increased in the order: fresh blood oranges < 100% BOJ < BOJ-based beverages. Hence, the consumption of fresh blood oranges represents the cheapest way to ensure the intake of health-promoting bioactive compounds such as anthocyanins and HCAs.

## Introduction

Blood oranges are considered a health-promoting food thanks to the pool of bioactive compounds characterizing their composition, with special regards for ascorbic acid, hydroxycinnamic acids (HCAs), and anthocyanins (Riso et al., 2005; Guarnieri et al., 2007). Anthocyanins are natural pigments determining the red color of many fruits and vegetables, such as cherries, grapes, currants, blackberries, strawberries, etc., which have demonstrated to behave as potent antioxidants in the human body (Riso et al., 2005), thus contributing to fighting various kinds of diseases (Prior and Wu, 2006; Wang and Stoner, 2008). Vitamin C represents the highest fraction of total antioxidant activity of orange juice, contributing, according to various studies, for about 60% (Gardner et al., 2000), 70% (Arena et al., 2001), up to 87% (Miller and Rice-Evans, 1997). Hydroxycinnamic acids (HCAs), the most widely distributed phenolics in plants, contribute in the diet as antioxidant and free-radical scavengers. They play a crucial role in the flavor alteration of blood oranges (Fallico et al., 1996) and their distribution, particularly ferulic, sinapic, p-coumaric, and caffeic, has been demonstrated to be a marker of Italian blood orange juices (Rapisarda et al., 1998). Hydroxycinnamic acids (HCAs), as well as other phenolic compounds have proved to be effective in the prevention of cardiovascular diseases and cancer, however, their efficacy depends on the level of intake and on their bioavailability.

Bioavailability of a nutrient involves many processes starting with the ingestion, including absorption, distribution, utilization and loss. It's defined as “the fraction of an ingested nutrient which may have the potential to meet functional demands in target tissues.” The mechanisms controlling both the tissues uptake and the relative distribution within the body of a bioactive compound are not clear (Davey et al., 2000). Although new studies are highlighting the limits of such an approach, to date, measuring the amount of an oral dose entering in plasma circulation has used to assess bioavailability of a specific compound. The plasma levels of a bioactive are strongly influenced, but not completely defined, by the dietary intake. The initial concentration, the form, the presence of other substances, associated with inter-individual variability, can affect the bioavailability of a bioactive compound. For instance, the form in which the ascorbic acid is taken may influence the absorption. Higher level may be absorbed when is taken with food, probably due to a longer gastrointestinal time (Davey et al., 2000). Ingestion of high doses of ascorbic acid (>200 mg/day) seems to have no advantage. In fact, it not significantly increases the absorption processes. The relative amount of absorbed ascorbic acid decreases from 100% at low doses, to about 80% at 180 mg, about 50% at 1.5 g, up to <16% at 12 g (Davey et al., 2000). The plasmatic concentration of ascorbic acid, in health fasting adults, ranges between 25 and 100 μmol/l. Although an upper limit has not been fixed, levels higher than 60 μmol/l seems to be enough to indicate tissues saturation (Graumlich et al., 1997). Even if ascorbic acid seems to affect different systems, the main action in biochemical systems appears to be related to its antioxidant capacity, being in charge of about the 15%, up to 30%, of the antioxidant capacity of plasma. Many studies carried out both *in vitro* and *in vivo*, report that the combination of dietary antioxidant vitamins and polyphenols may have synergistic effects on lipid peroxidation and antioxidant capacity (de Kok et al., 2008). Anthocyanins can exist in different forms under different pH values, changing visible color, residue charge, as well as solubility. So these molecules can be modified during food processing and/or storage, as well as after ingestion. Studies have shown very high antioxidant capacity, but low bioavailability (<1%) and concentration in plasma estimated at nanomols/nanograms (Yang et al., 2011). Food matrix may affect, synergistically or antagonistically, the bioavailability of anthocyanins. Studies *in vivo* showed that after 3 weeks of a diet rich in blood orange juice (600 mL/day), consumers had a significant increase of ascorbic acid, cyanidine-3-glucoside, and other compounds in plasma, but, no significant effect on markers of oxidative stress (Riso et al., 2005). Further studies (Vitaglione et al., 2007) identified protocatechuic acid (PCA) as one of the major metabolites of cyanidine-3-glucoside, explaining up to the 74% of the ingested anthocyanin from blood orange juice. Phenolic compounds showed different stability under gastric conditions. *p-*Coumaric acid increased its concentration, whereas ferulic and sinapic acids diminished and caffeic acid remained almost at the same concentration. Hydroxycinnamic acids showed a decrease (average 14%) under small intestinal digestion. Ferulic acid was the most bioaccessible phenolic acid (26%), followed by sinapic and p-coumaric (17%) and caffeic acid the lowest one (0%) (Rodríguez-Roque et al., 2013). Moreover, more work is needed on the fate of these compounds in the gastrointestinal tract and, especially, on the actual absorption level and metabolism (El-Seedi et al., 2012).

The consumption of red fruits has been recommended as an important contribution to healthy diets. A high number of red fruit-based products are available today, in response to the increased consumers demand health-promoting products. Juices and beverages claiming red orange among their composition gain higher market value, thanks to the demonstrated health-promoting potential of red orange bioactive components (Bitsch et al., 2004; Galvano et al., 2004; Bonina et al., 2005), however, their real nutritional value and contribution to a healthy diet remains uncertain. A previous study (Fallico et al., 2010) demonstrated that artificial colorants, rather than anthocyanins, contribute most to the red color of such products and that their nutritional quality is far from that of blood orange juices (BOJ), both for the low percentage and low quality of the red orange juice used. Nevertheless, juice-based beverages have recently increased their market share compared with 100% juices. The same authors (Fallico et al., 2011), assessing the exposure to Allura Red from the consumption of red-juice based drinks in Italy, reported that this category represents 5.7% of the total juice-based drinks market. These beverages have commonly a pleasant taste and flavor and usually reach a longer shelf life compared with 100% juices, this feature representing the counterpart of the lower bioactive content. Recently, Fallico et al. (2017) have reviewed the bioactive compounds levels in blood oranges and have calculated the contribution of blood oranges consumption to ascorbic acid, HCAs and anthocyanins daily intake: this article also highlighted that the industrial concentration processes of BOJ do not determine significant reductions in anthocyanins and HCAs. Only slightly (10%) reduce the vitamin C content. The same article pointed out that information is lacking on the actual nutritional value of BOJ-based drinks and on their impact on the intakes of bioactive compounds. Indeed, a literature survey confirmed that information on the bioactive content is widely available for fresh juices, while it is unavailable for BOJ-based commercial beverages: the only paper retrieved (Scordino et al., 2015) focused on the anthocyanin content of commercial red orange-based drinks, demonstrated that only 60% of the 50 samples analyzed had minimum content of anthocyanins from blood oranges, while absence was recorded for the remaining 40% of samples, whether due to degradation during shelf life or to fraud (i.e., non-use of BOJ).

No guarantee, thus, is given to the consumer about the nutritional value of these products as the legislation does not provide neither the indication of origin of the blood juice nor the minimum percentage of red juice that must be contained in the beverage to use the denomination “Blood orange,” nor the minimum level of bioactive compounds in the commercial beverages.

Therefore, the aim of this paper was to screen the levels of bioactive components, namely ascorbic acid, HCAs and total anthocyanins, in commercial beverages containing BOJ, to estimate their contribution to the intake of health-promoting compounds and finally discuss the actual value of the products on a price/bioactive level basis.

## Materials and methods

### Sampling and samples description

A total of 20 different brands of red beverage samples, two independent lots each, for a total of 40 elementary units were sampled. These representing all of the BOJ and BOJ based beverages present in 27 shops, which constitute about the 10% of food retailers in the metropolitan area of Catania (Italy) (about 1.0–1.5 million people). Samples were classified according to Codex General Standard For Food Additives (GSFA^1^) and FoodEx2 (EFSA, 2015) food category systems. The detail on the samples, including codification, % juice and blood orange juice (BOJ), ingredients and time to expiry date (at the moment of analysis) are reported in Table 1. For each sample, the determination of ascorbic acid, total monomeric anthocyanins and HCAs were performed in duplicate, as reported hereafter. The data reported in the tables are, therefore, the average of four independent analyses.

**Table 1:** Samples description.

**Sample**	**Replicate^a^**	**Days to expiry**	**% juice**	**% blood orange juice**	**Other juices**	**Healthy ingredients**	**Colorant**
AR01	1	13	100	100			
	2	33					
AR02	1	30	100	59	Grape 40%, elder 1%		
	2	180					
AR03	1	202	30	30	Blackcurrant and carrot extract		E 120
	2	278					
AR04	1	202	30	30		Vitamin C, anthocyanins	
	2	187					
AR05	1	344	30	30	Black carrot concentrated juice	Ascorbic acid (antioxidant)	
	2	233					
AR06	1	48	30	30	Blackcurrant and carrot extract		E 120
	2	206					
AR07	1	292	40	25	Blonde orange 15%	Vitamin C	
	2	279					
AR08	1	172	25	25	Blackcurrant and carrot extract	Vitamin C	E 120
	2	225					
AR09	1	202	20	20		β-Carotene, ascorbic acid (antioxidant)	
	2	150					
AR010	1	322	40	20	Orange 20%, black carrot and pomegranate	Vitamin C	
	2	323					
AR011	1	133	40	18.4	Apple 15%, pomegranate 6.4%, elder 0.2%	Vitamin C	
	2	135					
AR012	1	394	17	17	Orange, black carrot	Vitamin C	
	2	389					
AR013	1	234	25	15	Blonde orange 10%		
	2	300					
AR014	1	72	25	13	Orange 12%, pomegranate, black carrot	Vitamin C	
	2	205					
AR015	1 2	211 113	30	10	Apple, orange, lemon, pomegranate and carrot (tot. 20%)	Vitamin C, A, E	
AR016	1	150	30	9	Grape 13%, pomegranate 3%, cherry 5%	Vitamin C plus other nine vitamins	
	2	180					
AR017	1	157	20	8	Grape 7.5%, mandarin 4.5%	Ascorbic acid (antioxidant)	
	2	122					
AR018	1	189	30	8	Orange 15.3%, apple 6.7%	Ascorbic acid (antioxidant)	
	2						
AR019	1	180	31	4	Orange 27%	β-Carotene	
	2	173					
AR20	1	191	12.5	0.5	Orange 12%	Anthocyanins	
	2	192					

a *Replicates of each sample were from two different lot of production*.

### Vitamin C determination

L-ascorbic acid was determined by the 2,6-dichloroindophenol titration method, according to the official methods of the American Organization of Analytical Chemistry (AOAC Official Method 967.21., 2005a) as suitably modified by Rapisarda and Intelisano (1996) for the analysis of pigmented orange juices. Briefly, juice was centrifuged and paper-filtered, then 15 mL juice was passed through LC-18 cartridge (Supelco, 1,000 mg) previously activated with 3 mL methanol and washed with 5 mL water. The first 5 mL of sample was discarded, while the remaining sample was collected. Cartridges were regenerated with 10 mL HCl 0.1 M in methanol, followed by 5 mL acetone and 5 mL water. Two mL of clarified sample was added with 7 mL acid solution (HPO_3_-CH_3_COOH) and titrated with 2,6-dichloroindophenol solution until persistent pink. Results are expressed as mg of ascorbic acid/100 g of juice.

### Total anthocyanins determination

Total anthocyanins were determined according to the differential pH method (AOAC Official Method 2005.02., 2005b). Aliquots of 5 mL sample were diluted to 25 mL with a buffer solution at pH 1 (potassium chloride, 0.025 M) and pH 4.5 (sodium acetate, 0.4 M), respectively, and absorbance was read at 520 and 700 nm. Total monomeric anthocyanins were expressed as mg of cyanidin-3-glucoside equivalents/100 g of juice.

### Total hydroxycinnamic acids (HCAs) determination

HCAs were detected as reported by Rapisarda et al. (1998) with some modifications. Ten milliliters of clear juice was added to 10 mL of 2 N NaOH and stored at room temperature in the dark for 4 h, to hydrolyse the bound forms of HCAs. After, the solution was acidified with HCl 2 N to pH 2.5. The mixture after centrifugation at 10°C (13,000 rpm for 15 min) was filtered with a syringe PTFE filter (0.45 μm) (Albet) and injected into HPLC system (Shimadzu Class VP LC-10ADvp) equipped with a DAD (Shimadzu SPD-M10Avp). The column was a Gemini C-18 NX (150 × 4.6 mm, 5 μm) (Phenomenex), fitted with a guard cartridge packed with the same stationary phase. The HPLC conditions were according to Rapisarda et al. (1998). All the solvents used were HPLC purity grade. Standard solution, used for calibration curve, was prepared dissolving known amounts of commercial standards of caffeic, coumaric, ferulic and sinapic acid (Sigma-Aldrich, Milan, Italy) in acidified water (pH 2.5, with HCl).

### Estimation of bioactives intake from BOJ-based beverages consumption

The Estimated Daily Intake (EDI) of bioactive compounds (mg/day), per serving portion (125 g) and in long-time exposure, was determined according to (Douglass and Tennant, 1997). The concentration of each bioactive was multiplied by the serving portion or the daily consumption at the most probable (mean) and maximum (95th) percentile values, respectively. Data used for the estimation of daily consumption in long-time exposure were those concerning the most realistic scenario (3B) referred to BOJ consumers, reported by Fallico et al., (2011). This scenario considers data on beverages purchased in selected shops and the distribution of red juice-based drinks sales in 1 year. Moreover, it takes into account that each item is consumed by a number of consumers (family members, sons, parents, other) as declared by the buyer. The mean value of consumption for both BOJ and BOJ-based beverages was 18.9 mL/day up to 27.2 mL/day at the 95th percentile (Fallico et al., 2011).

### Statistical analysis

Levels of bioactive compounds (ascorbic acid, total anthocyanins, caffeic, coumaric, ferulic and sinapic acids, total HCAs and Σ bioactives) in the different samples were analyzed using one-way analysis of variance (ANOVA) followed by a *post-hoc* Tukey's test. When the variance was not homogeneous, data were analyzed using Games-Howell's test. *p* < 0.05 was defined as statistical significance.

To analyse the relationship between % of blood orange juice and level of ascorbic acid, total anthocyanins, total HCAs, level of each bioactive compounds and time to expiry, level of bioactive compounds and price of beverages Pearson correlation coefficients were calculated. All statistical analyses were performed with Minitab 18 (Minitab Ltd, Coventry, United Kingdom).

## Results and discussion

The composition, quality, as well as the product names such as fruit juices, fruit nectar and fruit-based drinks, are subject to specific legislation: the Codex Standard for Fruit Juices and Nectars (CODEX STAN 247, 2005) and, in the European Union, the Directive 2001/112/EC, recently amended by the Directive 2012/12/EU Council Directive 2001/112/EC, Directive 2012/12/EU. In particular, besides defining the characteristics of 100% juices, they define “citrus (orange) nectars” as the products containing at least 50% (v/v) orange juice. Firstly, considering the above legislation, samples were classified according to GSFA and FoodEx2 systems. Two samples were classified as fruit juices, one (AR01) was a pure BOJ, the other sample (AR02) contained 59% BOJ and 41% other juices (Table 1). None of the remaining 18 samples was classified as nectar, but all as a fruit-based beverage: in fact, these beverages contained from 12.5% up to 40% of fruit juices (Table 1). The trade name of all samples suggested blood oranges, even if the labeled % of BOJ ranged from 30 to 0.5%. The food classification system could be very useful for the understanding of the true nature of these beverages. As said above, all labels refer to fresh fruits, particularly to blood oranges, category 04.1.1 or A01CS after GSFA or FoodEx2 systems, respectively. Through the use of pictures/photos on the packaging and the reference to “fruit-based beverage,” producers suggest the consumer that the package contains a fruit juice (category 14.1.2.1 or A03AM, after GSFA or FoodEx2, respectively) or, at least, a fruit nectar (category 14.1.3.1 or A03BG, after GSFA or FoodEx2, respectively). However, with the exception of the two pure juices (AR01 and AR02), for all analyzed samples the right classification would be under the categories: 14.1.4.2– Non-carbonated water-based flavored drinks, after GSFA system, or A0FOJ– water-based beverages, in particular, A03FL– soft drink orange flavor, according to FoodEx2.

The appropriate classification of the products highlights that they are not actually fruit-based beverages but water-based ones; hence labels should more correctly mention “water-based” instead of “fruit-based.”

The addition, in the juice-based beverages, of red vegetable extracts and E120 suggests that the level of anthocyanins in the BOJ used was not enough to assure the red color. Artificial colorants were found responsible for about 91% of the color of commercial red juice-based drinks and red healthy drink (Fallico et al., 2010).

Ascorbic acid, being a potent antioxidant, is added to most of the beverages both as preservative and/or as a health bioactive compound. In the last case, according to art. Thirteen of Regulation EU 1924/2006 and Regulation EU 432/2012, the vitamin C addition could be claimed on labels and relative figures and contribution to daily intake should be given on the nutritional label (Regulation (EU) No 1169/2011, 0000)Regulation (EC) No 1924/2006,Commission Regulation (EU) No 432/2012.

### Ascorbic acid

Results show high variability in the ascorbic acid levels in BOJ-based beverages (Table 2). The ascorbic acid level in samples without exogenous ascorbic acid (AR01, AR02, AR03, AR06, AR13, AR19, AR20) ranged from < 1 to about 48 mg/100 g, with the highest figures for the 100% blood orange juices (AR01). The level of ascorbic acid in commercial orange juices (100%) ranged from about 74 mg/100 mL to about 35 mg/100 mL (Kabasakalis et al., 2000; Klimczak et al., 2007; Rodríguez-Bernaldo de Quirós et al., 2009), higher than those found in fruit beverages (about 20 mg/100 mL) and commercial cocktail of a mix of fruit juices (about 15 mg/100 mL) (Kabasakalis et al., 2000; Rodríguez-Bernaldo de Quirós et al., 2009). On the whole, the ascorbic acid level was not correlated with the percentage of BOJ in the beverage, and samples containing 25–40% juice, which were supplemented with ascorbic acid as antioxidant or nutraceutical ingredient, showed ascorbic acid levels which ranged from about 10 to about 67 mg/100 g. This trend points out the attempt of producers to give added value to products, which have a low nutritional value. Hence, the ascorbic acid level should not be taken as a quality marker of orange juice-based beverages since it is often intentionally added as an antioxidant and/or nutraceutical element and it is not possible to distinguish between its preservative or nutraceutical function. Even if the antioxidant activity level is surely beneficial for consumers, its recovery is not related to the genuineness of the product.

**Table 2:** Levels of bioactive components in BOJ-based beverages.

**Sample**	**Ascorbic acid**	**Total anthocyanins**	**Caffeic acid**	**Coumaric acid**	**Ferulic acid**	**Sinapic acid**	**Total HCA**	**Σ of Bioactives**
	**(mg/100 g)**	**(mg/100 g)**	**(mg/100 g)**	**(mg/100 g)**	**(mg/100 g)**	**(mg/100 g)**	**(mg/100 g)**	**(mg/100 g)**
AR01	48.2 ± 0.57^ac^	2.43 ± 0.20^ab^	0.51 ± 0.01^d^	2.30 ± 0.03^a^	0.37 ± 0.002^a^	0.60 ± 0.01^ab^	3.78 ± 0.04^a^	54.38 ± 0.73^abc^
AR02	19.20 ± 1.45^f^	2.39 ± 0.23^ab^	0.49 ± 0.10^d^	1.31 ± 0.11^b^	0.22 ± 0.01^b^	0.31 ± 0.05^cde^	2.33 ± 0.27^bcde^	24.01 ± 1.93^e^
AR03	10.9 ± 1.17^f^	0.37 ± 0.12^b^	0.65 ± 0.05^d^	0.96 ± 0.01^bc^	0.19 ± 0.002^bc^	0.35 ± 0.07^bcd^	2.15 ± 0.03^d^	13.38 ± 1.28^e^
AR04	21.6 ± 2.27^f^	0.30 ± 0.04^b^	2.14 ± 0.08^a^	0.67 ± 0.04^de^	0.20 ± 0.05^abc^	0.27 ± 0.13^abcde^	3.27 ± 0.28^ab^	25.27 ± 2.15^e^
AR05	66.63 ± 3.47^ac^	0.64 ± 0.29^b^	1.19 ± 0.11^cd^	0.75 ± 0.04^d^	0.15 ± 0.02^bc^	0.21 ± 0.01^de^	2.30 ± 0.18^de^	69.61 ± 3.92^a^
AR06	6.14 ± 0.18^f^	0.27 ± 0.11^b^	0.65 ± 0.05^d^	0.83 ± 0.06^cd^	0.16 ± 0.03^bc^	0.25 ± 0.04^de^	1.89 ± 0.17^de^	8.34 ± 0.13^e^
AR07	62.26 ± 2.26^ab^	*nd*	1.28 ± 0.05^cd^	0.73 ± 0.05^d^	0.22 ± 0.03^b^	0.33 ± 0.10^abcde^	2.56 ± 0.20^dbcd^	64.88 ± 2.36^ab^
AR08	66.98 ± 5.05^a^	0.09 ± 0.03^b^	0.54 ± 0.26^d^	0.73 ± 0.10^cde^	0.14 ± 0.03^bc^	0.26 ± 0.02^de^	1.66 ± 0.38^cde^	68.61 ± 5.37^a^
AR09	38.00 ± 3.16^cde^	*nd*	0.17 ± 0.001^d^	0.47 ± 0.02^e^	0.09 ± 0.01^c^	0.11 ± 0.05^e^	0.85 ± 0.07^d^	38.87 ± 3.12^cde^
AR10	23.36 ± 1.32^ef^	1.95 ± 0.05^ab^	1.60 ± 0.01^bc^	0.79 ± 0.01^d^	0.22 ± 0.01^b^	0.40 ± 0.03^ac^	3.01 ± 0.05^bc^	28.30 ± 1.23^e^
AR11	27.78 ± 6.42^bcdef^	1.72 ± 0.76^ab^	1.55 ± 0.48^abcd^	0.83 ± 0.09^cd^	0.12 ± 0.05^bc^	0.29 ± 0.10^bcde^	2.80 ± 0.54^abcd^	32.45 ± 6.12^bcde^
AR12	41.31 ± 0.17^ad^	1.04 ± 0.04^ab^	0.57 ± 0.04^d^	0.81 ± 0.01^d^	0.20 ± 0.005^b^	0.44 ± 0.03^ac^	2.02 ± 0.07^d^	44.40 ± 0.27^ad^
AR13	37.51 ± 7.83^abcdef^	2.70 ± 0.91^ab^	2.10 ± 0.21^ab^	0.65 ± 0.04^de^	0.23 ± 0.04^ab^	0.69 ± 0.09^ab^	3.67 ± 0.13^a^	43.83 ± 8.82^abcde^
AR14	16.38 ± 2.05^f^	0.25 ± 0.12^b^	1.49 ± 0.18^bcd^	0.56 ± 0.04^de^	0.15 ± 0.003^bc^	0.19 ± 0.02^be^	2.40 ± 0.23^bcde^	18.98 ± 2.39^e^
AR15	12.12 ± 1.88^f^	0.41 ± 0.24^b^	1.81 ± 0.22^abc^	0.55 ± 0.06^de^	0.13 ± 0.03^bc^	0.20 ± 0.08^cde^	2.69 ± 0.38^abcd^	15.31 ± 2.47^e^
AR16	22.00 ± 0.58^ef^	3.20 ± 0.38^a^	1.19 ± 0.02^cd^	0.94 ± 0.02^bc^	0.08 ± 0.002^bc^	0.22 ± 0.01^de^	2.44 ± 0.04^bde^	27.63 ± 0.16^e^
AR17	11.17 ± 0.41^f^	0.90 ± 0.04^ab^	0.28 ± 0.006^d^	0.23 ± 0.01^f^	0.05 ± 0.001^c^	0.29 ± 0.02^be^	0.85 ± 0.01^d^	12.91 ± 0.38^e^
AR18	10.81 ± 0.52^f^	0.03 ± 0.01^b^	0.30 ± 0.01^d^	0.58 ± 0.02^de^	0.10 ± 0.004^bc^	0.01 ± 0.0005^e^	0.99 ± 0.04^d^	11.84 ± 0.51^e^
AR19	18.26 ± 0.52^ef^	0.01 ± 0.01^b^	0.27 ± 0.12^d^	0.45 ± 0.17^cdef^	0.11 ± 0.07^abc^	0.06 ± 0.06^e^	0.89 ± 0.41^e^	19.03 ± 0.88^e^
AR20	0.63 ± 0.01^g^	0.32 ± 0.03^b^	0.46 ± 0.004^d^	0.39 ± 0.02^ef^	0.10 ± 0.003^bc^	0.11 ± 0.01^de^	1.05 ± 0.03^d^	1.99 ± 0.05^e^
Mean	28.06	0.95	0.96	0.77	0.16	0.28	2.18	31.20
St. dev.	19.96	1.05	0.64	0.42	0.07	0.17	0.92	20.34
Min5th	0.00	0.00	0.00	0.00	0.00	0.00	0.34	0.00
Max95th	67.98	3.04	2.25	1.61	0.31	0.62	4.02	71.88

Mean value ± standard deviation; data in column followed by different letter are significantly different at p < 0.05; * calculated as sum of ascorbic acid, total anthocyanins and total HCAs.

The comparison among different batches of the same brands suggests that ascorbic acid levels change with storage time. In most cases, samples belonging to batches closer to the expiry date had lower ascorbic acid, thus confirming this parameter as a marker of storage time even if no correlation was found between days to expiry and vitamin C levels.

Taking for granted the standardized composition of industrial beverages from different batches, these cases suggest very variable storage conditions occurring during the commercial life of beverages, which makes it difficult to predict the ascorbic acid concentration at consumption. Interestingly, the labeled ascorbic acid values in the samples claiming the supplementation with vitamin C were usually very different from the measured levels. In particular, most samples showed higher ascorbic acid concentration than declared: this could be explained with a prudential approach adopted by the producer, who might report in the label the minimum value that consumers would find at the end of shelf life or, as reported by Rodríguez-Comesaña et al. (2002), this is due to the fact that in label the amount refers only to the ascorbic acid added not considering the natural content of vitamin C of the juice. However, in three cases the ascorbate levels determined were lower than labeled, suggesting the need for more accurate prevision of its levels at consumption, and highlighting the occurrence of severe storage conditions at any stage of the distribution chain.

### Total anthocyanins

The anthocyanins level is the most important quality parameter for BOJ, which also determines the industrial price of such juices. As found for the ascorbic acid level, also the concentration of total anthocyanins varied significantly among samples (Table 2). The total anthocyanin values determined in the present study on the commercial 100% BOJs was 2.4 mg/100 g (Table 2). This concentration depends on many factors, such as the blood orange variety, degree of ripeness and season, anyway the anthocyanin level determined in the 100% commercial BOJs was much lower than the values reported in the literature for blood oranges and for industrial juices. According to literature, total anthocyanins range widely between 2.6 and 10.3 mg/100 g in freshly squeezed BOJ (Arena et al., 2001) and between 6.4 and 16.0 mg/100 g in blood oranges depending on the variety (Fallico et al., 2017). In processed BOJ, anthocyanins values ranged from 4.8 to 10.4 mg/100 g (Arena et al., 2001) and between 1.7 and 9.7 or 3.7 and 14.8 mg/100 g for NFC and RFC juices, respectively (Fallico et al., 2017). Moreover, it is widely accepted that the concentration processes of juices do not significantly affect the level of anthocyanins (Fallico et al., 1996, 2017; Arena et al., 2001).

In principle, the level of total anthocyanins should be a function of the percentage of BOJ and/or other red fruits included in the beverage formulation (i.e., blackcurrants, pomegranate, cherry, elder and black carrots). However, samples AR07 and AR09, claiming 25 and 20% BOJ, respectively, had no trace of anthocyanins. Taking as a reference the 100% BOJ sample (AR01), having a total anthocyanin content of 2.43 mg/100 g, samples containing 30% BOJ (not considering other anthocyanin-rich extracts), (AR03, AR04, AR05, and AR06), should have around 0.7 mg/100 g, while the actual figures ranged between 0.27 and 0.64. Similarly, the anthocyanin content for the sample containing 25% BOJ (AR08) and an unspecified addition of black currant extract was around 0.09 mg/100 g, much lower than expected (0.6 mg/100 g). On the other hand, total anthocyanins in samples AR10, AR11, AR12 and AR16, reporting only 20, 18.4, 17, and 9% of BOJ, respectively, and other red fruit juices or extracts, ranged between 1.04 and 3.20 mg/100 g. On the other hand, sample AR13, including 15% BOJ, had 2.7 mg/100 g anthocyanins, much higher than expected (0.37 mg/100 g); indeed, it can be estimated that the BOJ used for this beverage contained as high as 18 mg/100 g of anthocyanins. Analogously to ascorbic acid, no significant correlation was found between % of BOJ and anthocyanin levels.

Overall, some BOJ-based beverages showed low levels of anthocyanins, suggesting a poor quality of juices.

The comparison of samples from the same brands, but belonging to different batches, allowed to evaluate the effect of storage time and conditions on anthocyanins content. In most cases, the closest the expiry date, the lowest anthocyanin levels: indeed, in agreement with previous findings, anthocyanins undergo degradation during storage time, this reaction being temperature-dependant (Arena et al., 2001; Licciardello and Muratore, 2011). However, no correlation was found between days to expiry and anthocyanins level, and in four cases, the sample with a longer residual shelf life had a lower content of the typical pigments of BOJ (i.e., samples AR03, AR04, AR05, and AR07). Taking for granted the standardized composition of industrial beverages from different batches, these latter four cases suggest very variable storage conditions. A previous study conducted on commercial BOJ-based drinks highlighted the absence of characteristic anthocyanins in 40% of the samples (Scordino et al., 2015), hypothesizing a gradual degradation or a willful misconduct of producers: in any case, and according to our findings, the added value expected is not guaranteed to the consumer.

### Hydroxycinnamic acids (HCAs)

HCAs were found at higher concentrations in blood oranges compared to blonde ones, and their profile has been proposed as a marker of Italian blood orange juices (Rapisarda et al., 1998) according to these authors, the level of total HCAs in orange juices ranges between 5.7 and 12.2 mg/100 g depending on the orange variety. Fallico et al. (2017) reported total HCA values of 6.4, 8.7 and 9.9 mg/100 g for Tarocco, Sanguinello and Moro cultivars, respectively, and 8.3 and 8.4 mg/100 g for (NFC) and (RFC) juices. The sampled BOJ-based beverages showed highly variable levels of HCAs (Table 2), ranging from 3.8 mg/100 g for the 100% BOJ (sample AR01) to 0.85 mg/100 g, for sample AR09 containing 20% BOJ. Sample AR13 showed total HCAs comparable with 100% BOJ values, despite the low BOJ (15%) and blonde orange juice (10%) labeled level. The mean total HCA value calculated on BOJ-based beverages, not including the 100% juice, was 2.2 mg/100 g. In general, it was not possible to find a correlation between the % juice (total or BOJ) and HCAs, since other juices or extracts were often added. Moreover, the typical distribution and levels of HCAs in blood oranges could not be found in the investigated BOJ-based samples, this change probably due to the presence of extracts and juices from other species. Indeed, all beverages containing high HCA levels were not characterized by coumaric and/or ferulic acids, typical of BOJs, but by high levels of ferulic acid (Table 2). To sum up, the variability of data can be attributed both to the percentage and quality of the orange juice and to the presence, in the beverage formulation, or other juices or extracts; moreover, the percentage of BOJ in the beverage is not, by itself, an indicator of the health-promoting value of the product.

### Bioactives intake from BOJ-based beverages consumption

Fruit juices and juice-based drinks have gained popularity since they are considered as important sources of bioactive components. This belief might be only partially true. In fact, previous studies have raised concern on the low health-promoting potential of such class of beverages (Fallico et al., 2010), whose popularity takes advantage of the demonstrated beneficial properties of some constituents. In particular, it was highlighted that anthocyanins are responsible for a very low part of the red color, which is, in fact, often determined by synthetic food dyes (such as Allura Red, E129) (Fallico et al., 2011). The panorama has slightly changed in the last years, and synthetic dyes have often been replaced with natural colorants, such as carmine, or natural extracts rich of pigments with bioactive value (i.e., anthocyanins, carotenoids). Even if the trend in the use of dyes has changed, the aim pursued by producers remains the same: to sell products which are perceived by the consumer as health-promoting ones, through the reference to blood oranges in labels, having a low nutritional value (the relative concentration of BOJ can be as low as 0.5%).

Table 3 reports data concerning both the estimation per serving portion (125 g) and for a continue consumption (long exposure) at mean and at the highest intake values (95th percentiles), respectively. The first covers a short period up to 24 h; the second refers to the daily intake over the entire lifetime. These data are reported for ascorbic acid, total anthocyanins and HCAs and their sum (∑ of Bioactives), respectively. Moreover, two scenarios were considered: the first aimed to evaluate the effect of loyalty to a single brand, the second, regarding the general consumers, without loyalty to a specific brand.

**Table 3:** Estimated daily intake (mg) of bioactive compounds from BOJ-based consumption.

		**Ascorbic acid**		**Total anthocyanins**		**Total HCAs**		Σ **of bioactives**	
**Scenario**	**Sample**	**Per serving portion (125 g)**	**Long exposure^A^**	**Per serving portion (125 g)**	**Long exposure^A^**	**Per serving portion (125 g)**	**Long exposure^A^**	**Per serving portion (125 g)**	**Long exposure^A^**
Brand-loyal consumers	AR01	60.2	9.1 (13.1)	3.0	0.5 (0.7)	4.7	0.7 (1.0)	68.0	10.3 (14.8)
	AR02	24.0	3.6 (5.2)	3.0	0.5 (0.7)	2.9	0.4 (0.6)	29.9	4.5 (6.5)
	AR03	13.6	2.1 (3.0)	0.5	0.0 (0.1)	2.7	0.4 (0.6)	16.7	2.5 (3.6)
	AR04	27.0	4.1 (5.9)	0.4	0.0 (0.1)	4.1	0.6 (0.9)	31.5	4.8 (6.9)
	AR05	83.3	12.6 (18.1)	0.8	0.1 (0.2)	2.9	0.4 (0.6)	87.0	13.2 (18.9)
	AR06	7.7	1.2 (1.7)	0.3	0.0 (0.1)	2.4	0.4 (0.5)	10.4	1.6 (2.3)
	AR07	77.8	11.8 (16.9)	0.0	0.0 (0.0)	3.2	0.5 (0.7)	81.0	12.3 (17.7)
	AR08	83.7	12.7 (18.2)	0.1	0.0 (0.0)	2.9	0.3 (0.5)	86.7	13.0 (18.7)
	AR09	47.5	7.2 (10.3)	0.0	0.0 (0.0)	1.1	0.2 (0.2)	48.6	7.4 (10.6)
	AR10	29.2	4.4 (6.4)	2.4	0.4 (0.5)	3.8	0.6 (0.8)	35.4	5.4 (7.7)
	AR11	34.7	5.3 (7.6)	2.2	0.3 (0.5)	3.5	0.5 (0.8)	40.4	6.1 (8.8)
	AR12	51.6	7.8 (11.2)	1.3	0.2 (0.3)	2.5	0.4 (0.6)	55.5	8.4 (12.1)
	AR13	46.9	7.1 (10.2)	3.4	0.5 (0.7)	4.6	0.7 (1.0)	54.9	8.3 (11.9)
	AR14	20.5	3.1 (4.5)	0.3	0.1 (0.1)	3.0	0.5 (0.7)	23.8	3.6 (5.2)
	AR15	15.2	2.3 (3.3)	0.5	0.1 (0.1)	3.4	0.5 (0.7)	19.0	2.9 (4.2)
	AR16	27.5	4.2 (6.0)	4.0	0.6 (0.9)	3.1	0.5 (0.7)	34.6	5.2 (7.5)
	AR17	14.0	2.1 (3.0)	1.1	0.2 (0.2)	1.5	0.2 (0.2)	16.6	2.4 (3.5)
	AR18	12.9	2.0 (2.9)	0.0	0.0 (0.0)	1.2	0.2 (0.3)	14.1	2.2 (3.2)
	AR19	22.8	3.5 (5.0)	0.0	0.0 (0.0)	1.1	0.2 (0.3)	24.0	3.6 (5.2)
	AR20	0.8	0.1 (0.2)	0.4	0.0 (0.1)	1.3	0.2 (0.3)	2.5	0.4 (0.5)
General consumers	Mean	35.0	5.3 (7.6)	1.2	0.2 (0.3)	2.8	0.4 (0.6)	39.0	5.9 (8.5)

A *mg/day per capita mean value and at 95th percentile*.

Vitamin C intake coming from BOJ-based drinks is highly variable and, practically, depends on the ascorbic acid addition to beverages with an antioxidant or nutraceutical purpose. This value can be negligible or can represent a significant contribution to diet for high consumers of a specific brand (AR05, 07, 08). The dietary reference values for vitamin C depend on various factors, including age, sex and specific conditions (smoker, pregnancy, lactation): the (National Academy of Sciences et al., 2000), reported an Estimated Average Requirement (EAR) of 60 and 75 mg/day for young women and men, respectively; similarly, the EFSA NDA Panel (EFSA Panel on Dietetic Products (2013) reported a vitamin C Population Reference Intake (PRI) of 70 mg/day for women and men aged 11–14, which increased to 90 and 100 mg/day for the age range 15–17 and 95 and 110 mg/day for adult (>18 years old) women and men, respectively. A previous work focused on the intakes of bioactive compounds from blood orange consumption (Fallico et al., 2017) reported mean values for ascorbic acid ranging from 33 to 40 mg/day and from 53 to 64 mg/day for young and adult blood orange consumers, respectively, depending on the orange variety considered. Based on such data and on published Estimated Average Requirement (EAR) data for vitamin C, it was concluded that 50% of adult blood orange consumers receive more than 70 and 90% (for males and women, respectively) of the vitamin C EAR, while young blood orange consumers receive about 50%.

Data in Table 3 show that for general consumers the ascorbic acid intake form BOJ beverages is 35 mg/day per serving portion, 5.3 mg up to 7.6 mg/day in long-time exposure. The consumption of a given brand beverage (AR05, 07 and 08) can cover up to the 100% of vitamin C requirements, giving a daily intake of about 80 mg per serving portion and about 12 mg (16% of EAR) in long exposure assessment. Considering the results above and findings on the bioavailability of ascorbic acid from orange juice (Sanchez-Moreno et al., 2003; Riso et al., 2005) the level of ascorbic acid in some of the BOJ beverages could be enough to guarantee a meaningful increase of the plasmatic level of ascorbate. But, the same studies highlighted that the highest increases were measured after a certain number of days of regular consumption of the orange juice (7 up to 21 days). This means that some of these beverages in the present study can occasionally compensate for the shortcomings of diet. But, in long-term exposure, considering the low beverage daily intake, 18.9 mL (Fallico et al., 2011), they are practically unable to compensate vitamin C deficiency.

As concerns anthocyanins, although the absorption mechanisms are not completely understood, since they are excreted or absorbed at very low levels (<0.1%), all researches converge on the fact that the ingested doses are important factors in anthocyanins bioavailability (Cavalcante Braga et al., 2017). The consumption of BOJ-based beverages contributes to the daily intake of anthocyanins with about 1.2 or 0.2 mg considering the serving portion or the long exposure assessment, respectively. In the brand-loyal consumption scenario, with the exception of the two 100% juices (AR01 and AR02), only two BOJ-based beverages (AR13 and AR16) guarantee an intake of anthocyanins at the same level required to increase their concentration in plasma (Riso et al., 2005). But, these results, in the cited study, were achieved after 21 days of daily consumption of blood orange juice. The consumption of BOJ-based beverages for brand-loyal consumers ranges from no intake of anthocyanins (AR09 and AR19) up to a maximum of 0.6 mg/day per capita (AR16). Only seven brand samples supplied a higher anthocyanins intake with respect to the mean assumption of general consumers (0.2 mg/day per capita). Also, in this case, the comparison with the previous study (Fallico et al., 2017) highlights noticeable differences. The range of anthocyanins intake from blood orange consumption is 3.8–16.4 mg/day, depending on the orange cultivar: in particular, the mean intake for young and adult consumers is 6.3 and 8.5 mg/day, respectively, which is at least 7–9-fold higher than the intake arising from BOJ-based beverages consumption, as found in the present study. From such comparison, it emerges clearly that the consumption of BOJ-based beverages cannot be compared with that of blood oranges, as far as the intake of anthocyanins is concerned.

Fallico et al. (2017), focused on the bioactive intakes from blood orange consumption, highlighted that the intake of HCAs range from 6.3 to 9.8 mg/day depending on the variety, and that the mean intakes for young and adult consumers, based on different consumption patterns, are 5.4 and 8.5 mg/day, respectively. Considering data reported in Table 3, only the 100% blood orange juice (AR01) and four BOJ-based beverages (AR04, 10, 11, and 13) shall provide an intake of around 4 mg per serving portion. On the average, these beverages contribute to a daily intake of 2.8 mg per serving portion. In the long exposure scenario the intake was 0.2 up to 0.7 mg/day per capita for single brand consumers (AR09, AR17, and AR01, respectively) and 0.4 mg/day per capita for general consumers. The comparison with intake of HCAs from blood oranges strengthens the concept that the consumption of BOJ-based beverages should not be considered an alternative to blood oranges consumption, as far as the intake of bioactive compounds is concerned. The consumption of BOJ-based beverages contributes for as little to the HCAs intake.

The sum of bioactives compounds intake, per serving portion and in long-term exposure, shows a wide range from negligible, 2.5 and 0.4 mg/day per capita, up to about 35-fold higher (87 and 13.2 mg/day per capita), for consumers of specific brand (AR20 and AR05, respectively). Except for the 100% BOJ (AR01) the other samples with the highest intake of ∑ of bioactives (AR05, AR07, AR08) were those fortified with ascorbic acid. The consumption of blood oranges supplies an long-term intake as the sum of bioactives of 76.1 mg/day per capita (Fallico et al., 2017), a value about seven times higher than that found in the 100% BOJ (AR01). The majority of analyzed samples supply an intake of bioactives from 35 to 175 times lower respect to the one associated with blood oranges consumption.

The contribution of ascorbic acid to the overall bioactive intake was about 89%, while the contribution of the other bioactives was about 10%. This distribution seems to be different from those reported for blood oranges (Fallico et al., 2017). In the last case, the contribution was more balanced with a 77, 12.4, and 10.5% for ascorbic acid, total anthocyanins and HCAs, respectively. This suggests, one more, that the health value of the beverages is due to the addition of vitamin C and not to the characteristic pattern of bioactive compounds of blood oranges.

### Price-based considerations

Table 4 reports the average unitary cost (in €cent per mg) of bioactive components in BOJ-based beverage samples. A significant correlation (*R* = 0.645, *n* = 19; *p* < 0.01) was found between the price per mg of HCAs and that of anthocyanins, while no correlation exists among the price per mg of any of these two classes of bioactives with that of ascorbic acid (data not shown). This is likely due to the fact that ascorbic acid is often added to beverages as a low-value bioactive compound, whose addition as a preservative (antioxidant) is often exploited as a health-promoting factor. In detail, the cost per mg ascorbic acid is usually very low, ranging from 0.1 to 2 €cent, with one exception (AR20) recording the highest level (15.7 €cent/mg). The cost per mg HCA varied by about 10-fold, from 2.2 to 21.6 €cent/mg. Concerning anthocyanins, the relative cost ranged widely from 3.4 to 1,090 €cent/mg, with 100% BOJ sample recording the lowest levels (11.5 €cent/mg) compared with most other BOJ-based samples. The relative unitary cost of bioactives was also estimated for blood oranges, taking as a reference the average retail market prices for the season 2016–2017, i.e., 1.35 €/kg, and the levels of bioactives from Fallico et al. (2017). Ascorbic acid levels range between 50.6 and 61.2 mg/100 g in the main blood orange varieties; accordingly, a relative cost of 0.2–0.3 €cent/mg ascorbic acid can be inferred. As for anthocyanins, whose level ranges between 6.4 and 16.0 mg/100 g depending on the variety, the estimated cost varies from 0.84 to 2.11 €cent per mg. Concerning HCAs, Fallico et al. (2017) reported values ranging from 6.4 to 9.9 mg/100 g in blood oranges, hence, the estimated relative cost is 1.36–2.11 €cent/mg of HCA.

**Table 4:** Unitary cost (€cent/mg) of bioactive compounds from BOJ-based beverages (averaged across 19 samples), 100% BOJ and fresh blood oranges.

	**Ascorbic acid**	**Anthocyanins**	**HCAs**
	**Mean (min-max)**	**Mean (min-max)**	**Mean (min-max)**
BOJ-based beverages	1.52 (0.1–15.7)	129.27 (3.4–1,093)	7.58 (2.2–21.6)
100% BOJ	0.58	11.47	7.38
Blood oranges	0.22–0.27	0.84–2.11	1.36–2.11

Finally, the relative cost of the three classes of bioactive compounds considered, increased following the order: fresh blood oranges < 100% BOJ < BOJ-based beverages. The differences observed were especially marked for anthocyanins, which represent the most valuable components in blood oranges, whose unitary cost for 100% BOJ and BOJ-based beverages is about 8- and 88-fold higher than that of anthocyanins from fresh blood oranges.

## Conclusion

Blood oranges are widely regarded as a health-promoting product, and consumers might consider BOJ-containing beverages as beneficial for their health compared to other soft drinks: this study demonstrates, however, that this claim does not correspond to high levels of health-promoting compounds. The outcomes suggest that the reference to ingredients which have beneficial effects on human health, based on scientific evidence, might be misleading for consumers and would need regulation by the authorities, as it has been done for supplements and health-promoting ingredients added to foods and claimed in the labels. For this reason, accordingly to International Food Classification Systems, these beverages should refer in labels as “water-based beverage” instead of “fruit-based” ones.

This survey on BOJ-based beverages highlights that the claimed vitamin C values were often inconsistent with the actual concentrations. Unpredictable, unsuitable storage conditions could determine lower vitamin C levels than claimed, while producers adopting a prudential approach might report lower values than actual ones, as a guarantee of the minimum vitamin C available at consumption. In these regards, the study points out the need for more accurate labeling and for improvement in the distribution conditions.

Based on the findings of this study and on previous findings, the intake of bioactive components (ascorbic acid, anthocyanins and HCAs) from blood orange consumption is much higher compared to the consumption of BOJ-based beverages: this consideration should guide the choices of consumers targeting a healthy diet, suggesting that the consumption of blood oranges and 100% BOJs should be preferred, when possible, in order to guarantee the intake of a rich pool of bioactive compounds. Finally, the estimation of the cost per mg of bioactive component in BOJ-based beverages and the comparison with blood oranges, allows to conclude that the market value of BOJ-based beverages is not correlated with their real nutritional value in terms of bioactive compounds level, and that the consumption of fresh blood oranges, when possible, represents the cheapest way to ensure the intake of health-promoting bioactive compounds such as anthocyanins and HCAs.

## Author contributions

BF and EA conceived and designed the experiments. FL and VR performed the experiments. All authors contributed to the interpretation of the data and wrote the manuscript.

### Conflict of interest statement

The authors declare that the research was conducted in the absence of any commercial or financial relationships that could be construed as a potential conflict of interest.

## Footnotes

^1^http://www.fao.org/gsfaonline/foods/index.html (Accessed January 16, 2018).

## References

[B1] AOAC Official Method 2005.02 (2005b). Total monomeric anthocyanin pigment content of fruit juices, beverages, natural colorants, and wines. pH differential method first action 2005 in Official Methods of Analysis, of AOAC International, 18th Edn., *Revision 2, 2007* (Gaithersburg, MD: AOAC International), Chapter 37, 37.

[B2] AOAC Official Method 967.21 (2005a). Ascorbic acid in vitamin preparations and juices. 2,6-dichloroindophenol titrimetric method, 45.1.14 in Official Methods of Analysis, of AOAC International 18th Edn., Revision 2, 2007 (Gaithersburg, MD: AOAC International), Chapter 45, 22–23.

[B3] Arena E. Fallico B. Maccarone E. (2001). Evaluation of antioxidant capacity of blood orange juices as influenced by constituents, concentration process and storage. Food Chem. 74, 423–427. 10.1016/S0308-8146(01)00125-X

[B4] Bitsch R. Netzel M. Frank T. Strass G. Bitsch I. (2004). Bioavailability and biokinetics of anthocyanins from red grape juice and red wine. J. Biomed. Biotechnol. 5, 293–298. 10.1155/S1110724304403106 PMC1082893 15577192

[B5] Bonina F. P. Puglia C. Cimino F. Trombetta D. Tringali G. Roccazzello A. M. (2005). Oxidative stress in handball players: effect of supplementation with a red orange extract. Nutr. Res. 25, 917–924. 10.1016/j.nutres.2005.09.008

[B6] Cavalcante Braga A. R. Carisa Murador D. Mendes de Souza Mesquita L. Vera de Rosso V. (2017). Bioavailability of anthocyanins: gaps in knolwledge, challenges and future research. J. Food Compos. Anal. 68, 31–40. 10.1016./j.jfca.2017.07.031

[B7] CODEX STAN 247 (2005). Codex General Standard for Fruit Juices and Nectars. Rome: Food and Agriculture Organization.

[B8] Commission Regulation (EU) No 432/2012 of 16 May 2012 establishing a list of permitted health claims made on foods other than those referring to the reduction of disease risk and to children's development and health Text with EEA relevance. Official Journal of the European Union, L 136, 25.5.2012, 1–40.

[B9] Council Directive 2001/112/EC of 20 December 2001 relating to fruit juices and certain similar products intended for human consumption. Official Journal of the European Union L 010 , 12/01/2002, 58–66.

[B10] Davey M. W. Montagu M. V. Inzé D. Sanmartin M. Kanellis A. Smirnoff N. (2000). Plant L-ascorbic acid: chemistry, function, metabolism, bioavailability and effects of processing. J. Sci. Food Agric. 80, 825–860. 10.1002/(SICI)1097-0010(20000515)80:7<825::AID-JSFA598>3.0.CO;2-6

[B11] de Kok T. M. van Breda S. G. Manson M. M. (2008). Mechanisms of combined action of different chemopreventive dietary compounds. Eur. J. Nutr. 47, 51–59. 10.1007/s00394-008-2006-y 18458834

[B12] Directive 2012/12/EU of the European Parliament and of the Council of 19 April 2012 amending Council Directive 2001/112/EC relating to fruit juices and certain similar products intended for human consumption. Official Journal of the European Union L 115, 27/04/2012, 1–11.

[B13] Douglass J. S. Tennant D. R. (1997). Estimation of dietary intake of food chemicals in Food Chemical Risk Analysis (London: Chapman & Hall), 212–215.

[B14] EFSA NDA Panel (EFSA Panel on Dietetic Products Nutrition and Allergies) (2013). Scientific Opinion on dietary reference values for vitamin C. EFSA J. 11, 3418, 68 10.2903/j.efsa.2013.3418

[B15] El-Seedi H. R. El-Said A. M. A. Khalifa S. A. M. Göransson U. Bohlin L. Borg-Karlson A.-K.. (2012). Biosynthesis, natural sources, dietary intake, pharmacokinetic properties, and biological activities of hydroxycinnamic acids. J. Agric. Food Chem. 60, 10877–10895. 10.1021/jf301807g 22931195

[B16] European Food Safety Authority (EFSA) (2015). The Food Classification and Description System FoodEx2 (Revision 2). EFSA Supporting Publication. 10.2903/sp.efsa.2015.EN-804

[B17] Fallico B. Arena E. Chiappara E. Ballistreri G. (2010). Colour and label evaluation of commercial pasteurised red juices and related drinks. Food Addit. Contam. Part B Surveill. 3, 201–211. 10.1080/19393210.2010.525751 24779619

[B18] Fallico B. Ballistreri G. Arena E. Brighina S. Rapisarda P. (2017). Bioactive compounds in blood oranges (*Citrus sinensis* (L.) Osbeck): level and intake. Food Chem. 215, 67–75. 10.1016/j.foodchem.2016.07.142 27542451

[B19] Fallico B. Chiappara E. Arena E. Ballistreri G. (2011). Assessment of the exposure to allura red colour from the consumption of red juice-based and red soft drinks in Italy. Food Addit. Contam. Part A Chem. Anal. Control. Expo. Risk Assess. 28, 37–41. 10.1080/19440049.2011.596166 21854298

[B20] Fallico B. Lanza M. C. Maccarone E. Nicolosi Asmundo C. Rapisarda P. (1996). Role of hydroxycinnamic acids and vinylphenols in the flavor alteration of blood orange juices. J. Agric. Food Chem. 44, 2654–2657. 10.1021/jf9503319

[B21] Galvano F. La Fauci L. Lazzarino G. Fogliano V. Ritieni A. Ciappellano S.. (2004). Cyanidins: metabolism and biologicalproperties. J. Nutr. Biochem. 15, 2–11. 10.1016/j.jnutbio.2003.07.004 14711454

[B22] Gardner P. T. White T. A. C. McPhail D. B. Duthie G. G. (2000). The relative contributions of vitamin C, carotenoids and phenolics to the antioxidant potential of fruit juices. Food Chem. 68, 471–474. 10.1016/S0308-8146(99)00225-3

[B23] Graumlich J. F. Ludden T. M. Conry-Cantilena C. Cantilena L. R. Wang Y. Levine M. (1997). Pharmacokinetic model of ascorbic acid in healthy male volunteers during depletion and repletion. Pharmaceut. Res. 14, 1133–1139. 10.1023/A:1012186203165 9327438

[B24] Guarnieri S. Riso P. Porrini M. (2007). Orange juice vs vitamin C: effect on hydrogen peroxide-induced DNA damage in mononuclear blood cells. Br. J. Nutr. 97, 639–643. 10.1017/S0007114507657948 17349075

[B25] Kabasakalis V. Siopidou D. Moshatou E. (2000). Ascorbic acid content of commercial fruit juices and its rate of loss upon storage. Food Chem. 70, 325–328. 10.1016/S0308-8146(00)00093-5

[B26] Klimczak I. Malecka M. Szlachta M. Gliszczynska-Swiglo A. (2007) Effect of storage on the content of polyphenols, vitamin C the antioxidant activity of orange juices. J. Food Compost. Anal. 20, 313–322. 10.1016/j.jfca.2006.02.012

[B27] Licciardello F. Muratore G. (2011). Effect of temperature and some added compounds on the stability of blood orange marmalade. J. Food Sci. 76, C1094–C1100. 10.1111/j.1750-3841.2011.02335.x 22417545

[B28] Miller N. J. Rice-Evans C. A. (1997). The relative contributions of ascorbic acid and phenolic antioxidants to the total antioxidant activity of orange and apple fruit juices and blackcurrant drink. Food Chem. 60, 331–337. 10.1016/S0308-8146(96)00339-1

[B29] National Academy of Sciences (2000). Institute of Medicine Food and Nutrition Board (2000). Dietary Reference Intakes for Vitamin C, Vitamin E, Selenium, and Carotenoids. Washington, DC: National Academy Press.

[B30] Prior R. L. Wu X. (2006). Anthocyanins: structural characteristics that result in unique metabolic patterns and biological activities. Free Radic. Res. 40, 1014–1028. 10.1080/10715760600758522 17015246

[B31] Rapisarda P. Carollo G. Fallico B. Tomaselli F. Maccarone E. (1998). Hydroxycinnamic acids as markers of Italian blood orange juices. J. Agric. Food Chem. 46, 464–470. 10.1021/jf9603700 10554264

[B32] Rapisarda P. Intelisano S. (1996). Sample preparation for vitamin C analysis of pigmented orange juices. Ital. J. Food Sci. 3, 251–256.

[B33] Regulation (EC) No 1924/2006 of the European Parliament and of the Council of 20 December 2006 on nutrition and health claims made on foods. Official Journal of the European Union L 404, 30.12.2006, 9–25.

[B34] Regulation (EU) No 1169/2011 of the European Parliament and of The Council of 25 October 2011on the Provision of Food Information to Consumers Amending Regulations (EC) No 1924/2006 and (EC) No 1925/2006 of the European Parliament and of the Council, and Repealing Commission Directive 87/250/EEC, Council Directive 90/496/EEC, Commission Directive 1999/10/EC, Directive 2000/13/EC of the European Parliament and of the Council, Commission Directives 2002/67/EC and 2008/5/EC and Commission Regulation (EC) No 608/2004. Official Journal of the European Union, L 304, 22.11.2011, 18–63.

[B35] Riso P. Visioli F. Gardana C. Grande S. Brusamolino A. Galvano F.. (2005). Effects of blood orange juice intake on antioxidant bioavailability and on different markers related to oxidative stress. J. Agric. Food Chem. 53, 941–947. 10.1021/jf0485234 15713002

[B36] Rodríguez-Bernaldo de Quirós A. Fernández-Arias M. López-Hernández J. (2009). A screening method for the determination of ascorbic acid in fruit juices and soft drinks. Food Chem. 116, 509–512. 10.1016/j.foodchem.2009.03.013

[B37] Rodríguez-Comesaña M. García-Falcón M. S. Simal-Gándara J. (2002). Control of nutritional labels in beverages with added vitamins: screening of b-carotene and ascorbic acid contents. Food Chem. 79, 141–144. 10.1016/S0308-8146(02)00121-8

[B38] Rodríguez-Roque M. J. Rojas-Graü M. A. Elez-Martínez P. Martín-Belloso O. (2013). Changes in vitamin C, phenolic, and carotenoid profiles throughout *in vitro* gastrointestinal digestion of a blended fruit juice. J Agric. Food Chem. 61, 1859–1867. 10.1021/jf3044204 23374081

[B39] Sanchez-Moreno C. Cano M. P. de Ancos B. Plaza L. Olmedilla B. Granado F.. (2003). High-pressurized orange juice consumption affects plasma vitamin C, antioxidative status and inflammatory markers in healthy humans. J. Nutr. 133, 2204–2209. 10.1093/jn/133.7.2204 12840179

[B40] Scordino M. Sabatino L. Lazzaro F. Borzì M. A. Gargano M. Traulo P. (2015). Blood orange anthocyanins in fruit beverages: how the commercial shelf life reflects the quality parameter. Beverages 1, 82–94. 10.3390/beverages1020082

[B41] Vitaglione P. Donnarumma G. Napolitano A. Galvano F. Gallo A. Scalfi L.. (2007). Protocatechuic acid is the major human metabolite of cyanidin-glucosides. J. Nutr. 137, 2043–2048. 10.1093/jn/137.9.2043 17709440

[B42] Wang L. S. Stoner G. D. (2008). Anthocyanins and their role in cancer prevention. Cancer Lett. 269, 281–290. 10.1016/j.canlet.2008.05.020 18571839 PMC2582525

[B43] Yang M. Koo S. I. Song W. O. Chun O. K. (2011). Food matrix affecting anthocyanin bioavailability. Curr. Med. Chem. 18, 291–300. 10.2174/092986711794088380 21110799

